# A GAN-Based Self-Training Framework for Unsupervised Domain Adaptive Person Re-Identification

**DOI:** 10.3390/jimaging7040062

**Published:** 2021-03-25

**Authors:** Yuanyuan Li, Sixin Chen, Guanqiu Qi, Zhiqin Zhu, Matthew Haner, Ruihua Cai

**Affiliations:** 1 College of Computer Science and Technology, Chongqing University of Posts and Telecommunications, Chongqing 400065, China; liyy@cqupt.edu.cn (Y.L.); chensixin9868@gmail.com (S.C.); 2 Computer Information Systems Department, State University of New York at Buffalo State, Buffalo, NY 14222, USA; 3 College of Automation, Chongqing University of Posts and Telecommunications, Chongqing 400065, China; zhuzq@cqupt.edu.cn; 4 Department of Mathematics & Computer and Information Science, Mansfield University of Pennsylvania, Mansfield, PA 16933, USA; mhaner@mansfield.edu; 5 Computer Engineering Department, San Jose State University, San Jose, CA 95192, USA; ruihua.cai@sjsu.edu

**Keywords:** person re-ID, domain shift, style transfer, self-training

## Abstract

As a crucial task in surveillance and security, person re-identification (re-ID) aims to identify the targeted pedestrians across multiple images captured by non-overlapping cameras. However, existing person re-ID solutions have two main challenges: the lack of pedestrian identification labels in the captured images, and domain shift issue between different domains. A generative adversarial networks (GAN)-based self-training framework with progressive augmentation (SPA) is proposed to obtain the robust features of the unlabeled data from the target domain, according to the preknowledge of the labeled data from the source domain. Specifically, the proposed framework consists of two stages: the style transfer stage (STrans), and self-training stage (STrain). First, the targeted data is complemented by a camera style transfer algorithm in the STrans stage, in which CycleGAN and Siamese Network are integrated to preserve the unsupervised self-similarity (the similarity of the same image between before and after transformation) and domain dissimilarity (the dissimilarity between a transferred source image and the targeted image). Second, clustering and classification are alternately applied to enhance the model performance progressively in the STrain stage, in which both global and local features of the target-domain images are obtained. Compared with the state-of-the-art methods, the proposed method achieves the competitive accuracy on two existing datasets.

## 1. Introduction

Person re-identification (re-ID) as a crucial task in surveillance and security strives to retrieve the same people across multiple images captured by non-overlapping cameras or across multi-scene images captured by the same camera. Despite the great success in person re-ID, some limitations still exist in practical applications, such as the acquisition of high-quality feature representation, the domain shift between training and testing data, and the difficulty of model migration from source domain to target domain.

**Figure 1:**
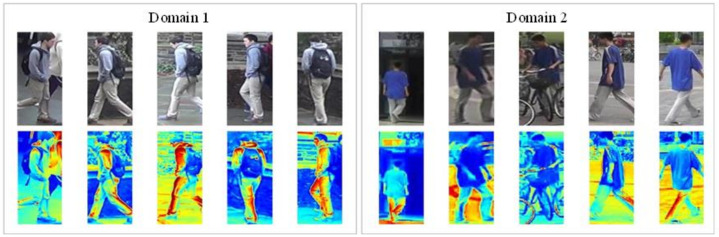
The sample images of person re-ID. The corresponding activation maps (AM) of the same pedestrians from two different domains as shown in the left- and right-hand sides, respectively.

Although existing person re-ID methods achieve high recognition rates on different types of single dataset, the great disparity exists between these person re-ID methods and practical applications, which is usually caused by the difference between the training and testing datasets [1,2,3,4,5,6,7,8,9]. As shown in Figure 1, different camera parameters, shooting conditions, and other factors cause the differences in exposure, image size, clarity, and other aspects. Therefore, if a model is trained on a single dataset according to the manually labeled data, the trained model often has poor recognition performance on real-world datasets.

The domain adaptive person re-ID was proposed to alleviate the domain shift issue between the labeled source domain and target domain. Some existing domain adaptive solutions achieve good performance in person re-ID. Existing domain adaptive person re-ID solutions can be roughly categorized into cross domain person re-ID [10] and shared domain learning person re-ID [11,12]. Specifically, cross domain solutions usually first conduct supervised learning on the labeled source domain, and then apply the learned model to the unlabeled target domain by migration learning. However, cross domain solutions ignore the global and local feature distribution of target-domain data, which is crucial for high-quality prediction. To compensate this deficiency, shared domain learning person re-ID solutions attempt to transfer the features in both source and target domains to a shared feature domain. Actually, due to the lack of supervisory signals during the transformation, it is difficult to guarantee the quality of visual feature information without enough constraints.

This paper proposes a GAN-based self-training framework with progressive augmentation (SPA) to solve the aforementioned two main challenges: the lack of labeled data and domain shift. This proposal expands the target-domain dataset by analyzing the consistency of each person identity in the labeled data and gains the pedestrian scale features in different learning strategies. Specifically, clustering and classification are applied to obtain the global and local features of each person respectively. The proposed SPA consists of style transfer stage and self-training stage, which act on the expanding and training processes respectively.

**Style transfer stage (STrans):** As a widely used style transfer solution in image processing, the proposed solution uses CycleGAN to achieve the transformation of the labeled image from source domain to target domain. Therefore, the generated training samples retain the style of target domain, such as resolution and light conditions. Specifically, Siamese network is applied to preserve the identities of pedestrians in the transformed images by using adversarial loss and contrastive loss. Besides, circle loss is used to mitigate the inflexibility and sensitivity of the proposed solution to image quality.

**Self-training stage (STrain):** Clustering and classification are integrated to learn the robust features of the unlabeled target domain. Therefore, the learned global and local features are semantically complementary. As the progressive augmentation learning, both global and local features of the target-domain data are gradually enhanced by alternate clustering and classification. Since clustering and classification can promote and supervise each other, the self-training process can be completed without external intervention.

The proposed solution applies a two-stage (STrans and STrain) method to data expanding and training. Source images are first transformed without distorting semantic contents, and then credible pseudo labels are generated. Therefore, the proposed solution can achieve good prediction performance. According to the comparative results, the proposed solution outperforms other state-of-the-art unsupervised domain adaptive person re-ID solutions on two benchmark datasets Market-1501 [13] and Duke-MTMC [14]. This paper has two main contributions as follows.

- A two-stage (STrans and STrain) framework is proposed for unsupervised domain adaptive person re-ID, which can achieve good performance on both image style transformation and self-training.
- A progressive augmentation learning strategy integrates clustering and classification to obtain both global and local features of the target-domain data, and generates credible pseudo labels without any interventions.

The rest of this paper is organized as follows. Section 2 introduces the related work; Section 3 presents the proposed image dehazing framework in detail; Section 4 discusses and compares the comparative experimental results; and Section 5 concludes this paper.

## 2. Related Work

As a critical task in intelligent monitoring, person re-ID that was first proposed by Gandhi in 2006 [15] has attracted considerable attention. Gray et al. [16] published a standard dataset called person re-ID VIPeR to test the performance of person re-ID solutions. Subsequently, the related person re-ID research boomed after the solutions of Zheng [17] and Farenzena [18] were published. Following the development of deep learning, person re-ID has achieved a significant breakthrough in both theories and applications. Existing solutions can achieve high recognition performance. Some recently published supervised person re-ID solutions have achieved more than 90% recognition rate on the relevant testing datasets, which greatly promote the development of the related applications.

**Figure 2:**
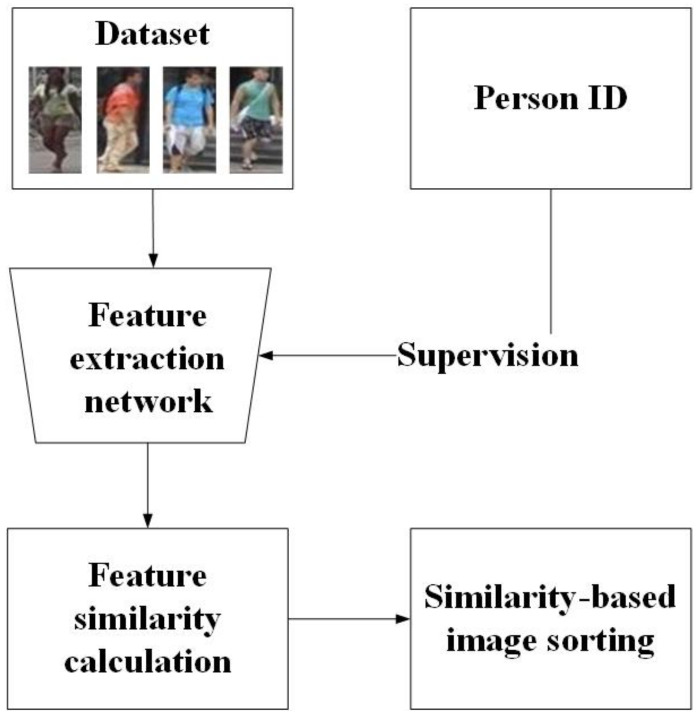
The general process of supervised person re-ID.

**Supervised person re-ID:** Supervised person re-ID methods conduct supervised training and testing on the same datasets [1,2,3,4,5,6,7,8,9,13]. As shown in Figure 2, the identity labels of pedestrian images are required. The features of the whole dataset are extracted by training the feature extraction network with the guidance of labels, which can be used to calculate the similarity between different images. According to the obtained similarity, the pedestrian images are sorted. A top ranked image contains highly similar features. Zheng et al. [19] explored how to use the generated data in training. Pedestrians are encoded as appearance and structure codes. Therefore, both self-identity and cross-identity people are generated, which make the dataset expansion become realistic. Considering both posture changes and unconstrained detection errors, a new joint learning method proposed by Li [20] integrates multi-scale attention selection and feature representation to maximize the relevant supplementary information of pedestrians.

Although existing supervised person re-ID methods can achieve good performance in the source domain, the lack of labeled samples and domain shift as two main issues still exist. Due to the difference of feature distribution between source and target domains, their recognition performance is often unsatisfactory in the target domain. Therefore, unsupervised domain adaptive (UDA) learning was proposed and applied to person re-ID to address the domain shift issue, which can be roughly categorized into cross domain learning and shared domain learning.

**Cross domain person re-ID:** Cross domain models can improve the object recognition accuracy, which are usually based on the supervised learning in the labeled source domain and applied to the unlabeled target domain by migration learning [21,22,23,24,25]. Peng et al. [24] proposed an unsupervised multi-task dictionary learning model, which represented the transferred visual features in unchanged visual angles from the source domain to the target domain. With the emergence and improvement of autoencoder, Potapov et al. [25] decomposed the interference variables of pedestrian images by potential coding, and a triple loss was used in the person feature extraction network. In addition, McLaughlin et al. [26] proposed a new data augmentation scheme based on the change of image background to alleviate the difference of data distribution caused by domain shift, which improved the cross-domain recognition ability.

**Shared domain person re-ID:** Shared domain-based person re-ID methods mainly focus on migrating the images in both source and target domains to a shared feature space [27,28,29,30]. In the shared domain, the consistency of visual feature information is preserved to solve the domain shift issue. To alleviate the dependence of existing methods on the labeled data, Li et al. [29] constructed a depth structure to project the features of different domains into the shared feature space by considering the labeled auxiliary dataset and the dataset of interest (without any label). In the process of shared domain person re-ID, the features from different domains are migrated to the shared feature space, and the similarity measurement of different images is realized in the shared feature space [30].

**Figure 3:**
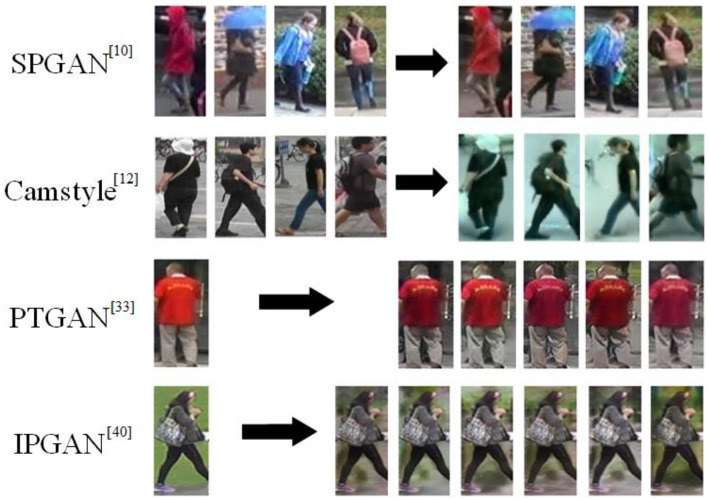
Transformed images obtained by different GAN-based person re-ID solutions.

**GAN-based person re-ID:** The acquisition and learning of valid datasets are two main steps of recognition. GAN [31] adopts the adversarial learning. Generator and discriminator can interact with each other in the process of adversarial learning, which are conducive to improving the recognition performance of person re-ID. Therefore, GAN-based person re-ID methods are booming. A similarity preserving generative adversarial network (SPGAN) proposed by Deng et al. [10] maintains the self-similarity and inter-domain differences to eliminate the domain shift by transforming the labeled samples from the source domain (called cycle-consistent generative adversarial networks (CycleGAN) [32]) to target domain. Inspired by CycleGAN, the Camstyle network proposed Zhong et al. [12] achieves the data augmentation by transferring the camera style of each image to different ones. Wei et al. [33] introduced the semantic segmentation of images to person re-ID and proposed the person transfer generative adversarial networks (PTGAN) to alleviate the domain shift issue between different domains. Figure 3 shows the transformed images obtained by different GAN-based person re-ID methods.

The robustness of UDA person re-ID methods is determined by the differentiated information from different domains. Due to the varying degrees of domain shift, the overall recognition performance of cross- and shared-domain person re-ID methods is not stable. Therefore, this paper explores how the labels, feature representation, and metric learning affect the performance of person re-ID and proposes an effective GAN-based self-training framework.

## 3. The Proposed Solution

**Figure 4:**
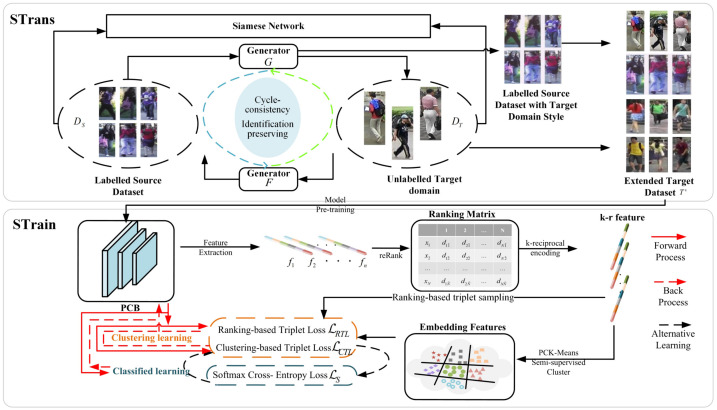
The framework of the proposed SPA.

As shown in Figure 4, the proposed SPA consists of STrans and STrain.

In Strans, both CycleGAN and Siamese Network are integrated to ensure the self-similarity (the same identity in an image is remained) and inter-domain difference (the original style is remained across different domains) before and after transformation. When any part of STrans is changed, the corresponding parameters of CycleGAN and Siamese Network are updated accordingly.

In Strans, the global and local structures of target-domain data are obtained in the two-stage self-training process of the progressive augmentation framework. In particular, the global and local features of each person are obtained by clustering and classification, respectively. Two stages process alternately in the self-training process until reaching the goal. Similarly, the corresponding parameters are updated according to any change of STrain.

### 3.1. Style Transfer Stage

**Figure 5:**
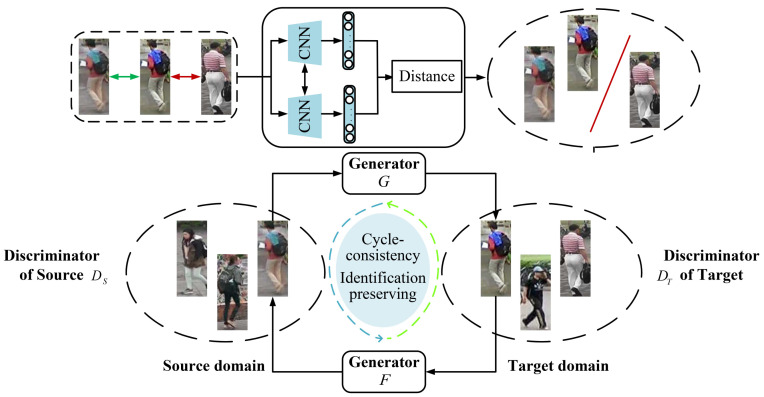
The structure of Strans (Style transfer stage).

Similar to SPGAN [10], CycleGAN [32] is used to realize the basic style transformation, and Siamese Network [34] is applied to maintain the consistency of pedestrian identity. Figure 5 illustrates the structure of Strans (Style transfer stage). As shown in the upper part of Figure 5, Euclidean distance is used to measure the similarity between two different images. The images with high similarity are clustered.

As shown in the lower part of Figure 5, CycleGAN learns generators *G* and *F* by capturing the fine information of the labeled source-domain dataset $S=\;\;\;\left\{\;\;\;x_{i}\;\;\;\right\}\;\;{\;}_{i=1}^{M}$ and unlabeled target-domain dataset $T=\;\;\;\left\{\;\;\;y_{j}\;\;\;\right\}\;\;{\;}_{j=1}^{N}$, respectively, which are used in the image style transformation from source domain to target domain. Adversarial loss and cycle-consistent loss are used to ensure the antagonism and consistency of image contents between *G* and *D*. CycleGAN is formalized as follows.

(1) $$\begin{array}{ccc} L_{cyc}\left(G,F,D_{T},D_{S}\right) & = & L_{adv}\left(G,D_{T}\right)+L_{adv}\left(F,D_{S}\right)\\ & & +\alpha L_{rec}\left(G,F\right) \end{array}$$

where $D_{T}$($D_{S}$) represents the discriminator corresponding to the generator *G*(*F*), $L_{adv}$ and $L_{rec}$ denote adversarial loss and cycle-consistency loss respectively, and α controls the relative importance of the cycle-consistent loss.

In addition to adversarial losses and cycle-consistency loss, style retain function is designed to ensure that the color composition between the input and output is preserved and the generator is prevented from outputting unreal results. In particular, when the generator transfers an image, it needs to preserve the identity information of source images. Therefore, a unit matrix is formed to ensure the identity mapping as follows.

(2) $$\begin{array}{ccc} L_{ide}\left(G,F\right) & = & E_{x^{s}∽p_{data}\left(S\right)}\left[{∥F\left(x^{s}\right)-x^{s}∥}_{1}\right]\\ & & +E_{x^{t}∽p_{data}\left(T\right)}\left[{∥G\left(x^{t}\right)-x^{t}∥}_{1}\right] \end{array}$$

It is necessary to ensure the identity consistency and domain dissimilarity of pedestrian after transformation. During the training process, Siamese network is optimized by minimizing the sum of contrastive loss and circle loss [35] on the designed input pair.

(3) $$\begin{array}{c} L_{con}\left(w,x_{1},x_{2}\right)=\frac{1}{2N}\sum_{n=1}^{N}\left(1-w\right){\left[\max(0,m-d)\right]}^{2}+w\cdot d^{2} \end{array}$$

where $\left(x_{1},x_{2}\right)$ is an input matching pair, *d* is the Euclidean distance between the pair, w=0 (w=1) denotes the input pair is negative (positive), and the parameter *m* controls the margin of decision boundary.

(4) $$\begin{array}{ccc} L_{circle} & = & \log[1+\sum_{i=1}^{L}\exp(\eta \alpha_{p}^{i}\left(d_{p}^{i}-\Delta p\right))\times \\ & & \sum_{j=1}^{K}\exp(-\eta \alpha_{n}^{j}\left(d_{n}^{j}-\Delta n\right))]LK \end{array}$$

where *L* and *K* represent the number of Euclidean distances corresponding to positive and negative input pairs respectively, L+K=N, and $d_{p}^{i}$ and $d_{n}^{j}$ denote the Euclidean distance between each matching pair. Due to the asymmetry of positive and negative pairs, Δp and Δn are the margin corresponding to them, respectively. η is used as an extended factor to realize the gradient control. To realize the self-paced weighting, $\alpha_{p}^{i}$ and $\alpha_{n}^{j}$ can be defined as follows.

(5) $$\begin{array}{c} \alpha_{p}^{i}={\left[O_{p}-d_{p}^{i}\right]}_{+}\\ \alpha_{n}^{j}={\left[d_{n}^{j}-O_{n}\right]}_{+} \end{array}$$

**Figure 6:**
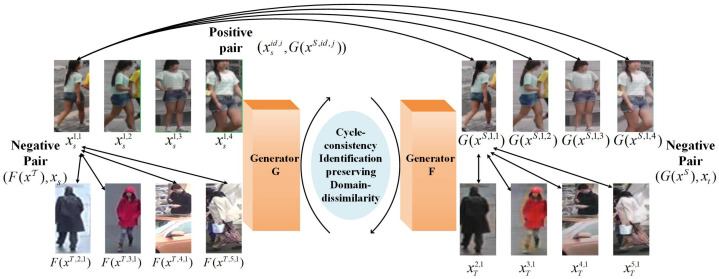
The image pair construction.

In Equations (3) and (4), loss functions use the binary labels of input image pairs. As shown in Figure 6, positive input pair $\left(x_{S}^{id,i},G\left(x^{S,id,j}\right)\right)$ and negative pair $\left(G\left(x^{S}\right),x_{t}\right)$ are designed to ensure the identity consistency and domain dissimilarity of pedestrians. Specifically, the i-th sample in source domain can be directly used to form a positive pair with any transformed image which has the same identity but not necessarily converted from the same sample. As the a priori knowledge that pedestrian images from two datasets do not cross and contain the same person, the pedestrians in the transformed images must be different from anyone from target domain. A negative pair is constructed as $\left(G\left(x^{S}\right),x_{t}\right)$ or $\left(F\left(x^{T}\right),x_{s}\right)$.

The overall objective function of style transfer stage can be formalized as follows.

(6) $$\begin{array}{ccc} L(G,F,D_{T},D_{S},M) & = & L_{cyc}\left(G,F,D_{T},D_{S}\right)+\lambda_{1}L_{ide}\left(G,F\right)\\ & & +\lambda_{2}L_{con}+\lambda_{3}L_{circle} \end{array}$$

The extended target domain dataset $T^{\prime }$ is obtained for further learning.

### 3.2. Self-Training Stage

Due to the dramatic appearance changes and identity dissimilarity between different domains, it is expensive and impractical to label data in the unsupervised and domain adaptation settings. To alleviate the above limitations, a two-step self-training process is proposed, which takes advantage of classification and clustering.

#### 3.2.1. Semi-Supervised Learning

**Figure 7:**
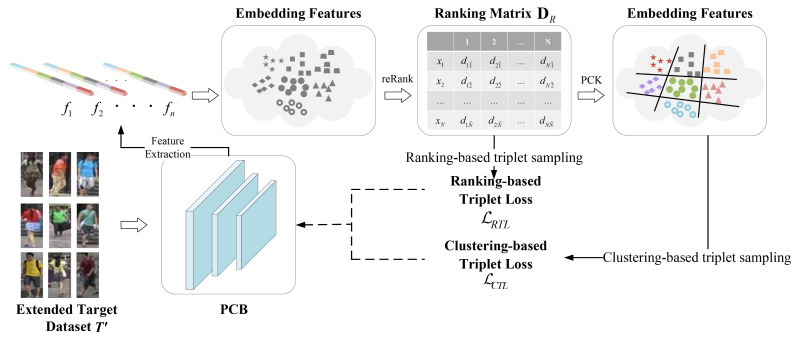
The semi-supervised learning structure. In semi-supervised learning, PCB first extracts the related features. Then k-reciprocal encoding is used to describe the fine difference between any two images, which yields a ranking score matrix $D_{R}$ for the next clustering operation. With the help of partially labeled images in the extended dataset $T^{\prime }$, semi-supervised clustering PCK-Means is used to mark pseudo-labels for the related data. The whole stage is trained by RTL and CTL.

Since the extended target domain dataset $T^{\prime }:\left\{t_{1},t_{2},\cdot \cdot \cdot ,t_{N^{\prime }}\right\}$ contains both true unlabeled samples (original target-domain images) and untrue labeled samples (converted from labeled source-domain images) after style transformation, semi-supervised learning is used to extract the embedding features from the pre-training part-based convolutional baseline (PCB) [36]. Subsequently, the pairwise constrained K-Means [37,38] (PCK-Means) is applied to semi-supervise sample clustering to obtain the reliable pseudo labels of untrue labeled samples. The semi-supervised learning structure is shown in Figure 7.

In practice, features $F:\{f\left(t_{1}\right),f\left(t_{2}\right),\cdots ,f\left(t_{N^{\prime }}\right)\}$ are extracted from the current PCB to construct the feature embedding space, and k-reciprocal encoding [39] is adopted to describe the fine difference between any two images. By calculating the Euclidean distance $d_{m}\left(t_{i},t_{j}\right)$ between the features of each pair, the neighbour set *N* corresponding to the k-closest distances of the probe is calculated. *N* that contains both positive and negative samples is defined as: $N\left(probe,k\right)=\left\{t_{1}^{0},t_{2}^{0},\cdots ,t_{k}^{0}\right\}$, where $t_{1}^{0},t_{2}^{0},t_{k}^{0}$ represent the 1st, 2nd, and k-th closest samples to the probe, respectively. At the same time, each $t_{i}^{0}$ of *N* also has its own neighbour set $N^{\prime }$. If a probe is included, probe and $t_{i}^{0}$ are adjacent to each other. Otherwise, they are not adjacent to each other. Thus, the k-reciprocal set *R* of the probe can be obtained, and all the elements in *R* are close to the probe. A ranking score matrix $D_{R}$ is obtained to describe the distance as follows.

(7) $$\begin{array}{ccc} D_{R} & = & {\left[D_{R}\left(t_{1}\right)D_{R}\left(t_{2}\right)\cdots D_{R}\left(t_{N}\right)\right]}^{T},\\ D_{R}\left(t_{i}\right) & = & \left[d_{J}\left(t_{i},{\tilde{t}}_{1}\right)d_{J}\left(t_{i},{\tilde{t}}_{2}\right)\cdots d_{J}\left(t_{i},{\tilde{t}}_{N}\right)\right]\\ & & \forall i\in \{1,2,\ldots ,N\} \end{array}$$

where $D_{R}\left(t_{i}\right)$ represents the ascending order of the distance between the probe $t_{i}$ and other samples in the gallery.

Given a large gallery, it is difficult to distinguish the samples with high similarity. PCK-Means is applied to mark pseudo labels for the extended target-domain dataset $T^{\prime }$, and the associated relationship in a mini-batch is explored to improve the operation speed of the proposed model in practical applications. In the end, P clusters and K instances are sampled randomly, and the cluster-based triplet Loss (CTL) is formulated as Equation (9).

(8) $$\begin{array}{ccc} L_{CTL} & = & \sum_{a=1}^{PK}{\left[m+||f\left(t_{a}\right)-f\left(t_{p}\right)|{|}_{2}-||f\left(t_{a}\right)-f\left(t_{n}\right)|{|}_{2}\right]}_{+}\\ & = & \sum_{i=1}^{P}\sum_{a=1}^{K}[m+\max_{p=1\ldots K}\;||f\left(t_{a}\right)-f\left(t_{p}\right)|{|}_{2}\\ & & -\min_{\begin{array}{c} n=1\ldots K\\ j=1\ldots P\\ i\neq j \end{array}}\;||f\left(t_{a}\right)-f\left(t_{n}\right)|{|}_{2}{]}_{+} \end{array}$$

where $\left(t_{a},t_{p},t_{n}\right)$ is a triplet, *m* is the margin between positive and negative pairs as same as Equation (3), and for the anchor $t_{a}$, *i* represents a certain class in *P* clusters, and *j* represents an instance under this class. Subsequently, benefiting from the PCK-Means, some samples could be added into $T_{U}$, which is the new image training set with pseudo labels to optimize PCB. However, it is clear that the effectiveness of CTL is highly subjected to the correctness of the clustering result. Hence, ranking-based triple loss (RTL) is proposed as follows, which does not depend on any pseudo labels, but is only related to the sorting matrix $D_{R}$.

(9) $$\begin{array}{ccc} L_{RTL} & = & \sum_{a=1}^{PK}[\frac{P_{p}-P_{n}}{\eta }m+||f\left(t_{a}\right)-f\left(t_{p}\right)|{|}_{2}\\ & & -||f\left(t_{a}\right)-f\left(t_{n}\right){\left|{|}_{2}\right]}_{+} \end{array}$$

where the triplet and parameter *m* are constructed in the same way as CTL, and for each anchor $t_{a}$, $P_{p}$ and $P_{n}$ represent the number of positive and negative pairs respectively. The combination of CTL and RTL can optimize the feature extraction network and capture the local information of data distribution effectively. Therefore, the final triple loss function in the semi-supervised learning can be defined as follows.

(10) $$\begin{array}{c} L_{C}=L_{RTL}+\lambda L_{CTL} \end{array}$$

where the parameter λ controls the relative importance of feature learning constraints.

#### 3.2.2. Classification Learning

Conventionally, according to the difference of objective loss function, person re-ID consists of representation learning and metric learning corresponding to classification and clustering respectively. Most existing methods use one way to train the network and the two learning methods are applied to further improve the network performance. Theoretically, due to PCK-Means clustering, the network focuses on the local structure of data distribution and may ignore the global information in semi-supervised learning. Therefore, the model is easy to fall into a sub-optimal local minimum.

As an optimization way, clustering and classification are used alternately. In this way, a fully connected layer is added to the end of the model as a classification layer, which is initialized by the current $T_{U}$. The objective function can be calculated by Softmax cross-entropy loss as follows.

(11) $$\begin{array}{c} L_{s}=\sum_{i=1}^{PK}\log\frac{e^{W_{{\hat{y}}_{i}}^{T}x_{i}}}{\sum_{c=1}^{C}e^{W_{c}^{T}x_{i}}} \end{array}$$

where ${\hat{y}}_{i}$ is the pseudo label of $x_{i}$, *C* denotes the cluster number of the updated training set $T_{U}$ after PCK-Means clustering, and *W* is the initialized classifier weight.

## 4. Comparative Experiments

### 4.1. Datasets and Objective Evaluation Indicators

Two large-scale person re-ID datasets as shown in Table 1, Market-1501 and Duke, are used to test the performance of the proposed model.

Market-1501 [13] is a dataset collected and published by Tsinghua University in 2015. 32,688 images were captured by six cameras including a low-definition camera, which involved 1501 pedestrians. Each pedestrian appeared in at least two camera views. Market-1501 is divided into training and testing sets, which have 12,936 images with 751 pedestrians and 19,732 images (including 3368 manually drawn images) with 750 pedestrians, respectively.

DukeMTMC-ReID [14] is a large-scale multi-pedestrian dataset collected by Duke University, which contains a large number of labels. Eight high-definition cameras collected 85-min video, involving 36,411 images and 1812 pedestrians. 1404 pedestrians appeared in at least two camera views. Zheng et al. [14] divided the dataset into the training set containing 1622 images with 702 people, testing query set containing 2228 images, and testing gallery set with 17,661 images. For convenience, Duke is short for DukeMTMC-ReID in the following paragraphs.

Cumulative matching feature (CMC) is the most widely used in person re-ID, which can be regarded as the accuracy rate in the related papers. For each pedestrian in the query set, it calculates the distance to *n* gallery samples in turn, and then sorts the obtained distances to check whether the same identity samples are located in the top-k, and finally the CMC curve is obtained by statistics. Specifically, it is a floating-point number in an interval. In convenience, it usually takes the form of percentage and only compares three-digits accuracy rates.

As an index widely used in reflecting the recall rate of the model, mean average precision (mAP) is the mean value of average accuracy (AP) of all query samples [40,41,42,43]. For the query sample probe, the calculation of its AP is mainly determined by the accuracy of recall rate. Specifically, AP of a query sample can be calculated as the area of precision-recall (PR) curve and horizontal axis.

### 4.2. Implementation

CycleGAN and Siamese network are adopted in the style transfer stage. Adam optimizer [44] is also used. The batch size is 1 and the initial learning rate is set to 0.0002. The training stops after the network has passes 6 epochs. Siamese network contains 3 convolutional layers (Con.), 3 maximum pooling layers (Max pooling), and 2 fully connected layers (FC). The specific network structure is shown in Table 2.

Similar to the EANet, PCB [36] is used as the feature extractor in the self-training stage. The feature tensor is horizontally divided into six parts to ensure the retention of local information. Deriving from numerous experiments and previous experiences, *m* in Equations (3) and (8), and α in Equation (1) are empirically set to 2 and 10, respectively. All input images are resized to 384 × 128 × 3. The dimension of each embedding layer is set to 256, the batch size is set to 64, and the number of iterations is set to 4.

The two-step learning rate can improve the learning performance of the progressive self-monitoring learning framework, rather than using the same learning rate directly in both self-monitoring stage and classification stage. Therefore, the false label guidance can be avoided. Specifically, in the semi-supervised learning, the learning rate of the backbone network is initialized to $1\times 10^{-4}$, and the learning rate of the embedded layer is $2\times 10^{-4}$. In the classification learning, the classification layer is $1\times 10^{-3}$, while all other layers are set to $5\times 10^{-5}$. After three iterations, all learning rates are multiplied by 0.1. The super parameter *m* is set to 2 which is consistent with Equations (3) and (8).

### 4.3. Comparisons with the State-of-the-Art Solutions

SPA proposed in this paper is compared with the state-of-the-art style transfer learning and UDA learning solutions on Market1501 [13] and DukeMTMC-reID [14]. Table 3 and Table 4 show the comparisons, in which M and D represent Market-1501 and Duke respectively. In each column, the highest result is marked in bold.

As shown in Table 3, transfer learning-based methods include Camstyle [12], PTGAN [33], SPGAN [10], IPGAN [45], MMFA [46], and UCDA [47]. PTGAN uses the semantic segmentation to constrain local images and retain the pedestrian information, but the direct conversion causes the loss of identity information easily. Camstyle, SPGAN, and IPGAN are all based on CycleGAN, which realize the unity of image styles between source and target domains. SPGAN and IPGAN use the identity retention to eliminate domain offsets, but they are limited by the matching pair construction methods. UCDA uses the transfer learning to minimize the invariance in target domain. STrans obtains 65.4 and 59.3 on Market and Duke of Rank-1, respectively, which benefit from the novel effective method to construct the matching pairs and optimize the model through circle loss with the target convergence.

As shown in Table 4, unsupervised methods include LOMO [48], BOW [21], PUL [49], BUC [50], DBC [51], PCB [36], and MAR [52]. LOMO and BOW use the hand-crafted features, which show low performance. MAR adopts the idea of multi-soft labeling. PCB is a baseline commonly used in recent research, which uses the horizontal division of high-dimensional tensors to retain the detailed information. TFsuion uses the spatio-temporal information to estimate the matching probability through Bayesian inference. However, the framework proposed in this paper is much more concisely and effective than existing methods. As shown in Table 4, mAP reaches 53.35% and 52.43% and rank-1 reaches 73.93% and 65.18% in D⟶M and M⟶D, respectively.

It is useful to use the expanded labeled data to train the model in the last two rows of Table 4. Specifically, compared with the style transfer stage alone, the incremental self-monitoring learning framework in rank-1 and mAP can improve by 3.86% and 3.07% in D⟶M, respectively.

### 4.4. Ablation Study

**The impact of the each component of the proposed algorithm.** As mentioned in introduction, the accuracy of person re-ID in UDA setting replies on the generation quality and identity recognition accuracy. Four components of the proposed GAN-based self-training network are evaluated as follows.

- GAN-based transformation network: According to the SPGAN model, the performance of the proposed GAN-based transformation network is significantly improved, which benefits from the adoption of both novel training data construction and circle loss methods.
- Progressive self-training framework: The semi-supervised clustering and classification learning are combined to learn the robust features of the unlabeled target domain effectively.
- Semi-supervised learning: k-reciprocal encoding and PCK-Means are used when a ranking score matrix is constructed and the initial images are clustered.
- Classification: It is identical to general softmax classification but needs to initialize the classification layer.

As show in Table 5, when the network only contains STrans, the rank-1 accuracy on M⟶D and D⟶M increases by 18.43% and 12.48%, respectively. The rank-1 and map of M⟶D increase by 20.53% and 12.47% respectively, while the rank-1 and map of D⟶M increase by 12.75% and 5.50% respectively. The improvement of efficiency shows that both triple losses can be used to enhance the performance of the proposed model, but the performance of STrans is slightly lower than that of STrain. STrans and STrain are combined to jointly optimize the model at the self-monitoring stage, and they achieve good results in M⟶D and D⟶M. Compared with STrans only, 1.71% and 3.40% improvements on rank-1 and map are achieved on D⟶M. Therefore, it confirms that a powerful target-domain feature extraction model is learned by the proposed SPA.

**Figure 8:**
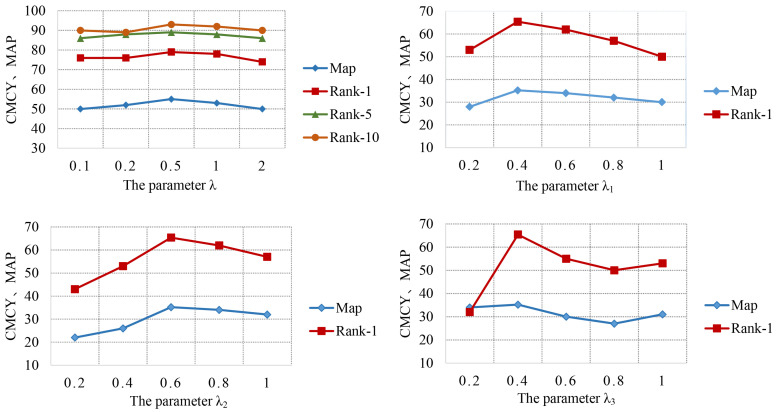
Analysis of parameters λ in Equation (10) and $\lambda_{1}$, $\lambda_{2}$, $\lambda_{3}$ in Equation (6).

**The impact of the hyperparameters.** The generalization properties of a loss function are governed by a small number of hyper-parameters. The hyperparameter values are determined in the process of model selection. In Equation (10), λ is used to control the weight between RTL and CTL. Values are selected from the set of 0.1, 0.2, 0.5, 1.0, and 2.0 to test the impact on the D⟶M task. When λ is low, RLT plays a major role, which tends to cause the overall network relying on the ranking score matrix $D_{R}$. Particularly, when the feature representations are in poor quality, the network performance is really low. When λ is high, the pseudo-label quality is low in the early stage of clustering process, and the network emphasizes the clustering results. As shown in the top left of Figure 8, the best result is obtained, when λ set to 0.5. However, the change in the size limits the performance improvement.

Subsequently, three hyperparameters in Equation (6) of STrans are tested, and the results are shown in the top right, bottom left, and bottom right of Figure 8. $\lambda_{1}$, $\lambda_{2}$, and $\lambda_{3}$ control the degree of style retain loss, contrastive loss, and circle loss respectively, which balance the impact of the losses and change from 0.2 to 1.0. When $\lambda_{1}$, $\lambda_{2}$, and $\lambda_{3}$ are set to 0.4, 0.6, 0.4, the best performance is achieved. When $\lambda_{1}$, $\lambda_{2}$, and $\lambda_{3}$ continually increase, a obvious drop occurs.

## 5. Conclusions

This paper proposes a GAN-based self-training framework for UDA person Re-ID, which focuses on solving the lack of pedestrian identification labels in the captured images and domain shift issue between different domains.

In the proposed SPA, the a priori knowledge from the labeled source domain is used to obtain the robust features of target domain. In style transfer stage, CycleGAN and Siamese Network are combined to ensure the self-similarity and inter-domain difference of person identification. Besides the widely used adversarial loss and contrastive loss, which are inflexible and sensitive to the quality of pair, circle loss is used to optimize the model with a targeted convergence. The self-training stage captures the global and local structure of target-domain data in the progressive augmentation framework, which takes advantage of clustering and classification on person re-ID. The comparative experimental results confirm the proposed solution achieves better performance than the state-of-the-art unsupervised cross-domain re-ID solutions in person re-ID. In future, the proposed method will be extended to other unsupervised cross-domain applications.

## Figures and Tables

**Table 1:** The details of person Re-ID datasets (Market-1501 and DukeMTMC-ReID).

Dataset	Publication Time	Number of IDs	Number of Cameras	Number of Images	Labeling Method	Image Size
Market-1501 [13]	2015	1501	6	32,688	Manual + DPM	128 × 64
DukeMTMC-ReID [14]	2017	1812	8	36,411	Manual	Random

**Table 2:** The design details of the STrans network structure.

Layer	Property	Parameter
1	Conv.1	4 × 4, stride = 2, fmap = 64
2	Max pooling 1	2 × 2, stride = 2,
3	Conv.2	4 × 4, stride = 2, fmap = 128
4	Max pooling 2	2 × 2, stride = 2,
5	Conv.3	4 × 4, stride = 2, fmap = 256
6	Max pooling 3	2 × 2, stride = 2,
7	Max pooling 4	2 × 2, stride = 2,
8	FC	Out: 256

**Table 3:** Person re-ID performance comparison with the state-of-the-art solutions of style transfer on Market-1501 and DukeMTMC-reID.

Method	D⟶M				M⟶D			
	Rank-1	Rank-5	Rank-10	mAP	Rank-1	Rank-5	Rank-10	mAP
Camstyle [12]								
PTGAN [33]	38.6	-	66.1	-	27.4	-	50.7	-
SPGAN [10]	51.5	70.1	76.8	22.8	41.1	56.6	63	22.3
IPGAN [45]	56.4	75.6	82.5	25.6	46.8	62	67.9	25.7
MMFA [46]	56.7	75	81.8	27.4	45.3	59.6	65	23
UCDA [47]	64.3	-	-	34.5	55.4	-	-	36.7
Ours	**65.4**	**80.6**	**85.7**	**35.2**	**59.2**	**72.8**	**76.7**	**37.6**

**Table 4:** Person re-ID performance comparison with the state-of-the-art solutions of unsupervised on Market-1501 and DukeMTMC-reID.

Method	Market-1501				Duke			
	Rank-1	Rank-5	Rank-10	mAP	Rank-1	Rank-5	Rank-10	mAP
LOMO [48]	27.2	41.6	49.1	8	12.3	21.3	26.6	4.8
BOW [21]	35.8	52.4	60.3	14.8	17.1	28.8	34.9	8.3
PUL [49]	45.5	60.7	66.7	20.5	30	43.4	48.5	16.4
PCB [36]	57.7	-	-	29.01	42.73	-	-	25.7
BUC [50]	66.2	76.6	84.5	38.3	47.4	62.6	68.4	27.5
DBC [51]	69.2	**83**	87.8	41.3	51.5	64.6	70.1	30
MAR [52]	67.7	81.9	-	40	**67.1**	79.8	-	48
Ours	**73.93**	82.8	**91.5**	**53.35**	65.18	**81.3**	**87.2**	**52.43**

**Table 5:** Ablation study on different components of the proposed method.

Method	Training	Market-1501		DUKEMTMC-reID	
		Rank-1	mAP	Rank-1	mAP
PCB	-	59.74	41.93	39.38	49.69
PCB+Style transform	Unsupervised	70.09	50.28	62.09	49.59
PCB+Self-training	Semi-supervised	71.22	51.85	63.49	48.43
PCB+SPA	Semi-supervised	73.93	53.35	65.18	52.43

## Data Availability

Not applicable.
